# The Genome-Wide Impact of *Nipblb* Loss-of-Function on Zebrafish Gene Expression

**DOI:** 10.3390/ijms21249719

**Published:** 2020-12-19

**Authors:** Marco Spreafico, Eleonora Mangano, Mara Mazzola, Clarissa Consolandi, Roberta Bordoni, Cristina Battaglia, Silvio Bicciato, Anna Marozzi, Anna Pistocchi

**Affiliations:** 1 Department of Biotecnologie Mediche e Medicina Traslazionale, Università degli Studi di Milano, LITA, Via Fratelli Cervi 93, Segrate, 20090 Milano, Italy; marco.spreafico@unimi.it (M.S.); mara.mazzola@unimi.it (M.M.); cristina.battaglia@unimi.it (C.B.); anna.marozzi@unimi.it (A.M.); 2 Institute of Biomedical Technologies, Italian National Research Council (ITB-CNR), Via Fratelli Cervi 93, Segrate, 20090 Milano, Italy; eleonora.mangano@itb.cnr.it (E.M.); clarissa.consolandi@itb.cnr.it (C.C.); roberta.bordoni@itb.cnr.it (R.B.); 3 Department of Life Sciences, University of Modena and Reggio-Emilia, Via G. Campi 287, 41125 Modena, Italy; silvio.bicciato@unimore.it

**Keywords:** *NIPBL*, RNA sequencing, zebrafish, gene expression regulation, acute myeloid leukemia

## Abstract

Transcriptional changes normally occur during development but also underlie differences between healthy and pathological conditions. Transcription factors or chromatin modifiers are involved in orchestrating gene activity, such as the cohesin genes and their regulator *NIPBL*. In our previous studies, using a zebrafish model for *nipblb* knockdown, we described the effect of *nipblb* loss-of-function in specific contexts, such as central nervous system development and hematopoiesis. However, the genome-wide transcriptional impact of *nipblb* loss-of-function in zebrafish embryos at diverse developmental stages remains under investigation. By RNA-seq analyses in zebrafish embryos at 24 h post-fertilization, we examined genome-wide effects of *nipblb* knockdown on transcriptional programs. Differential gene expression analysis revealed that *nipblb* loss-of-function has an impact on gene expression at 24 h post fertilization, mainly resulting in gene inactivation. A similar transcriptional effect has also been reported in other organisms, supporting the use of zebrafish as a model to understand the role of Nipbl in gene regulation during early vertebrate development. Moreover, we unraveled a connection between *nipblb*-dependent differential expression and gene expression patterns of hematological cell populations and AML subtypes, enforcing our previous evidence on the involvement of *NIPBL*-related transcriptional dysregulation in hematological malignancies.

## 1. Introduction

The capability of regulating gene expression is a key feature of cells and is implicated in all cellular processes. Thousands of genes are differentially transcribed within diverse cell types and whole-genome changes in gene expression constitutively occur during organism development and tissue differentiation. This transcriptional specificity depends on several regulatory proteins, such as transcription factors and chromatin modifiers, which determine time- and tissue-specific gene expression patterns through activation and repression of genes at genome-wide level. Diverse transcriptional patterns characterize not only physiological conditions but are also a distinct hallmark of pathological states. Thus, unrevealing factors involved in gene expression regulation and identifying their target genes might not only improve the knowledge of physiological development and tissue differentiation but also be exploited to design new therapeutic approaches for pathologies.

Cohesin is a ring-shaped multiprotein complex, which was initially identified as a key regulator of sister chromatid cohesion, segregation during mitosis [1], and genome stability maintenance during DNA repair [2]. The core structure of the cohesin complex is formed by the structural maintenance of chromosomes (SMC) subunits SMC1 and SMC3, by the α-kleisin subunit RAD21 and by a stromal antigen (SA) subunit, either SA1 or SA2. In addition, several proteins, such as ESCO1/2 acetylases and histone deacetylase 8 (HDAC8), are associated with the complex and regulate its function. In particular, the Nipped B-like (NIPBL) loading factor is required for cohesin loading onto the DNA. In fact, even though the complex itself is capable of binding the DNA [3], cohesin loading onto chromosomes requires *NIPBL* to be efficient. Apart from its aforementioned role, cohesin has recently emerged as an epigenetic regulator of gene expression. Studies have revealed that cohesin mediates long-range chromatin interactions between enhancers and promoters [4,5,6], and a recent work highlighted its role in the formation and stabilization of Topologically Associating Domains (TADs) [7]. In addition, there is evidence of cohesin-mediated regulation of gene expression, either positive or negative, through interaction with RNA polymerase II [8,9,10].

Since NIPBL is involved in mediating cohesin function, it also regulates cohesin-mediated gene expression. In particular, NIPBL co-localizes with cohesin and mediator at transcriptionally active sites [4]. Interestingly, NIPBL is also found to bind at the promoters of active genes in the absence of cohesin and its knockdown results in reduced gene expression of these genes [11]. Thus, *NIPBL* seems to play an even greater role than cohesin in regulating genome-wide gene expression. Indeed, previous works using different models highlighted *NIPBL* importance in transcriptional regulation during development and its deregulation was reported to alter the whole genome transcription and induce pathological defects, particularly in the Cornelia de Lange Syndrome (CdLS) models. Mutations in the *Nipped-B* gene, the *Drosophila melanogaster* ortholog of *NIPBL*, caused developmental defects in mutant flies [12] and *Nipbl*^+/-^ mice displayed alteration of multiple organs, including heart, bone, and fatty tissue [13]. Moreover, *Nipbl* deficiency impaired pectoral fin and limb development in zebrafish and mice respectively, through the down-regulation of several *Hox* genes [14]. In lymphoblastoid cell lines (LCLs) derived from CdLS patients carrying *NIPBL* mutations, cohesin-binding sites were reduced by approximately 30%, leading to transcription deregulation of more than 300 genes [15]. Similarly, transcriptome analysis revealed hundreds of deregulated genes, either down-regulated or overexpressed, both in iPSCs and cardiomyocytes derived from *NIPBL*^+/-^ CdLS patients [16]. And another study also identified whole-genome changes in gene expression in both LCLs derived from *NIPBL*-mutated CdLS patients and mouse embryonic fibroblasts (MEFs) derived from *Nipbl^+/−^* mice [17].

Other works associated *NIPBL* alterations with tumors. For example, the loss of *NIPBL* reduces sensitivity to chemotherapy in gastrointestinal cancers [18] and its mutation was found in acute megakarioblastic leukemia associated with Down syndrome (DS-AMKL) [19]. Interestingly, we have observed modulation of the canonical Wnt pathway in zebrafish embryos at different developmental stages upon *nipblb* loss-of-function: at 24 h post fertilization (24 hpf) we reported a reduction of canonical Wnt pathway genes [20], that was recovered starting from two days post-fertilization (dpf) [21]. This evidence raises the possibility that *nipblb* depletion primes a series of back-up mechanisms that counteract the transcriptional patterns induced by its silencing.

In this work, we further analyze the transcriptional role of *nipblb* loss-of-function in zebrafish embryos broadening the analyses to the whole transcriptome through RNAseq analyses. In this regard, we considered a possible role of *nipblb* choosing to compare wild-type and *nipblb* deficient embryos at 24 hpf. The experiments allowed the identification of strong *nipblb*-mediated gene inactivation in early development. Moreover, functional categories of affected genes are similar to those found in other *NIPBL*-depleted organisms, enforcing the rationale of using zebrafish as a model to unravel the role of *NIPBL* in gene regulation and diseases. In addition, we confirmed our previous findings highlighting the role of *NIPBL* in the regulation of pathways common to hematopoiesis and mutated *NPM1*.

## 2. Results

*NIPBL* has emerged as a major player in gene expression regulation at the genome-wide level and its alterations have been reported to underlie substantial transcriptional changes, particularly in CdLS. In this work, we studied the role of the *NIPBL* orthologue in zebrafish, *nipblb* (Ch 10: 36,475,860–36,547,128), in transcriptional activation relatively to a specific developmental stage. To assess the global *nipblb* impact on the whole-genome transcription during zebrafish development, we performed RNA-seq analysis on three replicates of control- and *nipblb*-morpholino (*nipblb*-MO, Gene Tools) injected embryos at 24 hpf (ctrl-MO 24 h and *nipblb*-MO 24 h). For *nipblb* specific knockdown we co-injected two non-overlapping morpholinos: the ATG and 5′UTR morpholinos.

**Figure 1:**
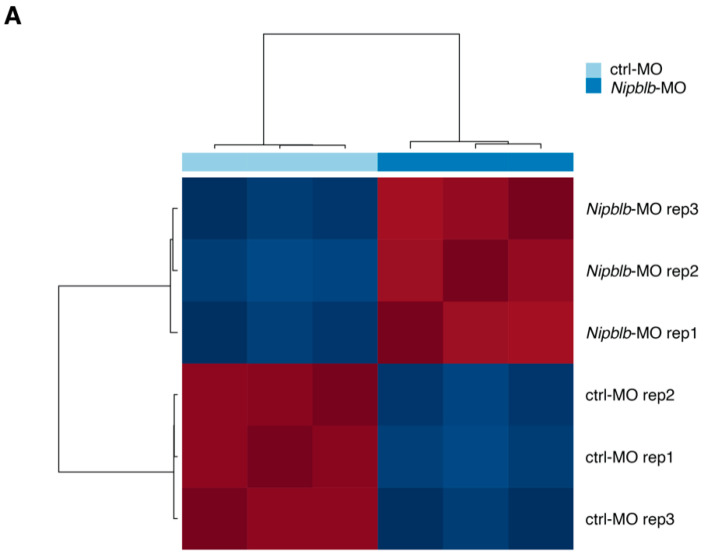

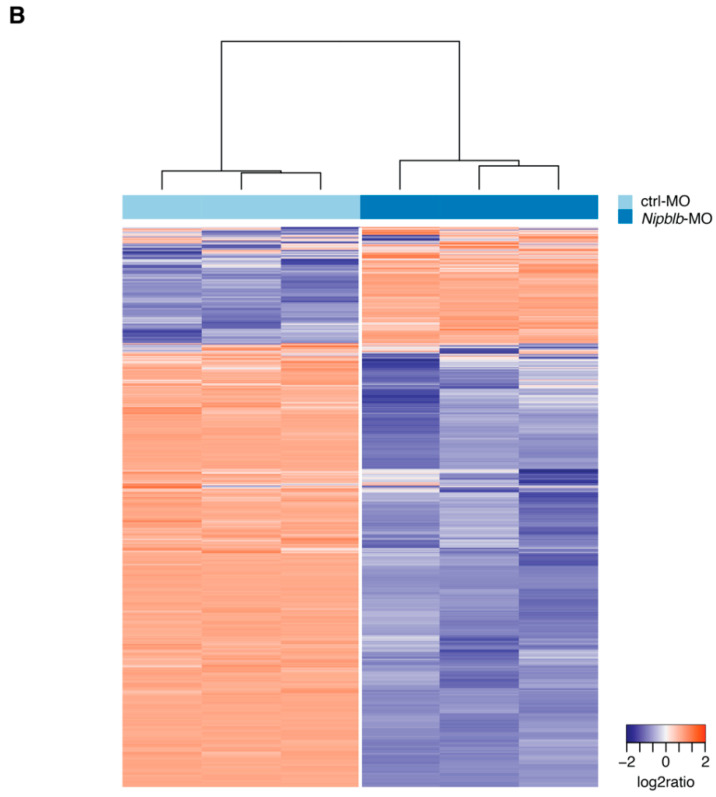
(**A**) Sample correlation matrix using gene-wise standardized expression values of 1922 highly variable genes. (**B**) Unsupervised hierarchical clustering of ctrl-MO and *nipblb*-MO-injected embryos at 24 hpf based on the standardized gene expression values of 1922 highly variable genes. Each column represents one separated biological sample.

For the generated six libraries we obtained, on average, 84 million reads with a range from 95.8% to 96.5% of reads mapped onto zebrafish genome (Table S1). We chose this developmental stage since at 24 hpf the overall structure of the body has already been completed and the three main axes are assessed following gastrulation and somitogenesis. The unsupervised analysis highlighted that the gene expression data were highly reproducible among the biological replicates (Figure 1A) and that *nipblb*-MO-injected embryos at 24 hpf were characterized by a completely distinct transcriptional pattern as compared to control embryos (Figure 1B).

**Figure 2:**
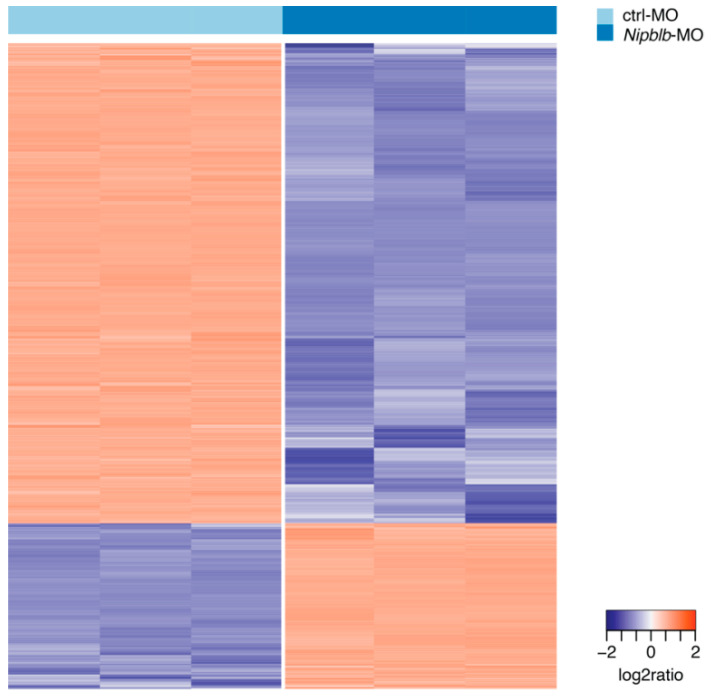
Hierarchical clustering of ctrl-MO and *nipblb*-MO-injected embryos based on the standardized gene expression values of 5338 genes differentially expressed in the comparison between *nipblb*-MO and ctrl-MO embryos at 24 hpf. Each column represents one separated biological sample.

To explore the molecular changes underlying loss of *nipblb* in early developmental stages and the interplay between *nipblb* loss-of-function and development, we compared the transcriptional profiles of zebrafish embryos from the two conditions (Table S2). Differential gene expression analysis returned 5338 genes differentially expressed in the comparison between *nipblb*-MO and control embryos at 24 hpf (false discovery rate FDR ≤ 1% and absolute fold change ≥ 3; Figure 2). Among them, 1366 genes were up-regulated and 3972 were down-regulated.

**Figure 3:**
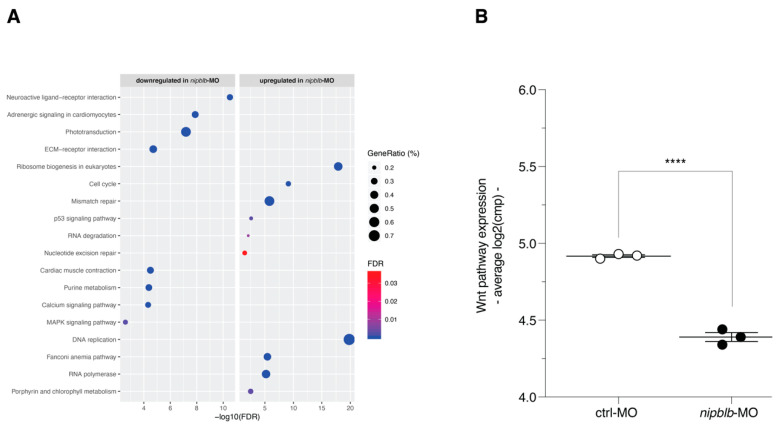
(**A**) Dot plot of the KEGG gene sets significantly enriched in the 3972 down-regulated (left) and in the 1366 up-regulated (right) genes by *nipblb* loss-of-function at 24 hpf. Dot color indicates statistical significance of the enrichment (false discovery rate FDR); dot size represents the fraction of genes annotated to each term. Gene sets are ranked in decreasing order based on the FDR value. (**B**) Average expression of KEGG Wnt canonical pathway genes in sample subgroups (**** *p* ≤ 0.0001 in unpaired *t*-test).

Functionally, down-regulated genes resulted involved in pathways related to receptors and ligands associated with intracellular and extracellular signaling, phototransduction, purine metabolism, and MAPK signaling (Figure 3A; Table S3). Conversely, up-regulated genes were enriched in DNA replication, ribosome biogenesis, cell cycle, and RNA synthesis and degradation (Figure 3A; Table S3). As we previously showed, *nipblb* loss-of-function determined, at 24 hpf, a significant reduction in the overall activation of the Wnt canonical pathway [20,21] (*p* < 0.0001; Figure 3B).

**Figure 4:**
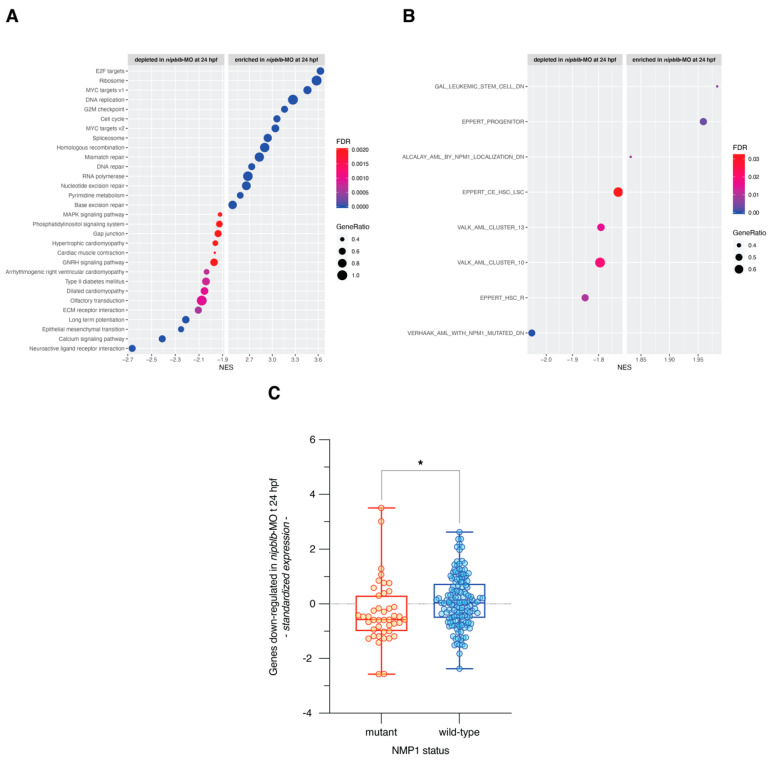
(**A**) Dot plot of the top 15 significantly enriched (normalized enrichment score NES > 0 and FDR ≤ 0.05) and depleted (normalized enrichment score NES < 0 and FDR ≤ 0.05) gene sets from Gene Set Enrichment Analysis of *nipblb*-MO at 24 hpf as compared to their ctrl-MO counterpart. Dot color indicates statistical significance of the enrichment (false discovery rate FDR), dot size represents the fraction of genes annotated to each term. Gene sets are ranked in decreasing order based on the NES value. (**B**) Dot plot of the significantly enriched (normalized enrichment score NES > 0 and FDR ≤ 0.05) and depleted (normalized enrichment score NES < 0 and FDR ≤ 0.05) myeloid differentiation and AML-related gene sets from Gene Set Enrichment Analysis of *nipblb*-MO at 24 hpf as compared to their ctrl-MO counterpart. (**C**) Average expression in *NMP1* mutant and wild-type AMLs of 3972 genes down-regulated by *nipblb* loss-of-function at 24 hpf (* *p* = 0.016 in unpaired *t*-test).

Functional annotation using GSEA and gene sets from the MSigDB Hallmark and KEGG collections highlighted that *nipblb* loss-of-function activated gene sets related to cell cycle, MYC signaling, DNA replication, RNA polymerase, and ribosome metabolism. On the contrary, it repressed gene sets associated with receptors and ligands involved in the intracellular and extracellular signaling, EMT, ECM, motility and function of the cardiac muscle, MAPK signaling pathway, and cell-matrix adhesion (Table S4 and Figure 4A).

Since *NIPBL* has emerged as a potential player in myeloid cell differentiation and in the insurgence of hematological malignancies [21,22,23], we performed the functional enrichment analysis also using a collection of gene sets related to myeloid differentiation and acute myeloid leukemia (AML; Table S5). Interestingly, we found that a set of genes up-regulated in human hematopoietic lineage-committed progenitor cells, as compared to hematopoietic stem cells (HSC) and mature cells, were significantly activated in *nipblb*-MO embryos at 24 hpf. Conversely, we found depleted sets of genes up-regulated in HSC enriched populations and in AML stem cell (LSC; Figure 4B and Table S6). In addition, *nipblb* loss-of-function down-regulated genes active in AML patients with wild-type *NPM1*, thus suggesting that *nipblb* knockdown phenocopies the transcriptional effects induced by *NMP1* mutations in AMLs (Figure 4B; Table S6). Next, we sought to determine the expression patterns of genes down-regulated by *nipblb* loss-of-function in a cohort of AML patients with mutant and wild-type *NPM1* [24]. We verified that the transcriptional program repressed by *nipblb* depletion is indeed repressed also in AMLs with mutant *NPM1* (*p* = 0.016; Figure 4C).

## 3. Discussion

The Nipped B-like protein (NIPBL) is the factor that allows the loading of the cohesin complex on the DNA during the G2 phase of the cell cycle [25]. In addition to its canonical role, NIPBL was also identified as a transcription factor and architect of the chromatin, independently of its interactions with the cohesin complex. For instance, Hi-C data showed that Nipbl deletion in murine cells strongly impacts genome organization, while few changes were reported following cohesin depletion [5,26,27]. Moreover, ChIP-sequencing analyses in human HB2 cells revealed that the binding sites of NIPBL to the genome were independent of those of cohesin or CTCF [11], suggesting that NIPBL may have a direct role in gene expression. Indeed, in HB2 cells, NIPBL was shown to localize preferentially to active promoters rich in CpG islands, and near the RNA Pol II binding sites [11]. Also in *D. melanogaster* genome, Nipped-B and cohesin preferentially bind to transcribed regions, frequently overlapping with RNA polymerase II binding sites [8]. In human ES cells, ChIP-Seq data revealed an association between NIPBL and the enhancer and core promoter region sites bound by mediator and cohesin in actively transcribed genes [4].

NIPBL has been linked to gene expression regulation in different models such as *Cut* and *Ultrabithorax* in *D. melanogaster* [28], adipogenic differentiation genes, such as *Cebpb* and *Ebf1* in murine embryonic fibroblasts [13], endodermal differentiation and left-right axis genes, such as *sox32*, *sox17*, *foxa2*, *gata5*, *spaw*, *lefty2*, and *dnah9* in zebrafish [29], histone deacetylases *HDAC1* and *HDAC3* [30] and heterochromatin protein 1 in human [31].

Here, we used RNA-seq analyses in *nipblb* loss-of-function zebrafish embryos generated through the injection of two morholino oligomers targeting the *nipblb* paralog. We chose this technique, already reported by our group and other authors [20,21,32], as a stable knock-out of cohesin genes is either lethal as homozygotes or dramatically compensated for in heterozygotes. Moreover, organisms may activate a compensatory network to buffer against deleterious mutations stably induced in their genome. These effects are particularly efficient when the genes are duplicated or there are different components of the same family that may compensate the mutant [33], as in the case of zebrafish *nipbl* paralogs. We investigated the genome-wide effect of *nipblb* loss-of-function on zebrafish transcriptional programs during development at 24 h post-fertilization. Our findings revealed a significant gene inactivation following down-regulation of *nipblb* in zebrafish embryos, as already described in *D. melanogaster* [8]. Indeed, as compared to their control samples, *nipblb* deficient embryos at 24 hpf showed a down-regulation of 3972 genes while only 1366 genes were up-regulated.

Among the down-regulated genes, we identified genes that could be correlated with phenotypes presented by CdLS patients with *NIPBL* mutation. For instance, we described down-regulation of genes associated with receptors and ligands involved in the intracellular and extracellular signaling, EMT, ECM, cardiac muscle motility and function, MAPK signaling pathway and cell-matrix adhesion. In a *Nipbl^+/-^* murine model, genes involved in neural differentiation, such as *Map2k1* and *Map4k1*, and heart development, such as *Bmp2,* were also described [13]. Interestingly, differences in gene expression were also described in iPSCs and cardiomyocytes derived from CdLS *NIPBL*^+/-^ patients, suggesting a role for *NIPBL* in transcriptional regulation of cardiac cells [16]. The functional enrichment of differentially expressed genes also resulted in pathways that were up-regulated following *nipblb* knockdown, such as MYC and JAK-STAT signaling pathways, and genes sets connected to interferon and immune response. Noticeably, the up-regulation of stress-induced immune response has also been described in *Nipbl^+/-^* MEF cells as a consequence of the down-regulation of RNA-processing genes and aberrant RNA biogenesis [17].

In recent years, dysregulation of *NIPBL* has been associated with tumor insurgence, in particular, in hematological malignancies [21,22,23]. To further verify this association, we used a collection of gene sets related to myeloid differentiation and AML in the functional enrichment analysis of the gene expression pattern induced by *nipblb* loss-of-function. Interestingly, we found that genes activated by *nipblb* knockdown are associated with the hematopoietic lineage-committed progenitor cells, while genes repressed by the loss of *nipblb* are expressed by HSC enriched populations and in AML stem cell (LSC). In addition, the down-regulation induced by *nipblb* loss-of-function resembles the transcriptional pattern induced by *NMP1* mutations in AMLs, as verified by the functional annotation and by the expression level of genes down-regulated by *nipblb* loss-of-function in the transcriptomes of AML patients with mutant and wild-type NPM1.

Taken together, these data provide new insights into the role of *nipbl* in regulating zebrafish gene expression. In particular, our analysis reveals that *nipblb* loss-of-function has a major impact on gene expression in the early development, until 24 hpf, and that this massive transcriptional dysregulation is significantly recovered later during development by compensative mechanisms that need further identification analyses. Moreover, by unraveling a connection between *nipblb*-dependent differential expression and gene sets related to different hematological cell populations and AML subtypes, we shed light on the possible involvement of *NIPBL*-related transcriptional dysregulation in hematological malignancies.

## 4. Materials and Methods

### 4.1. Zebrafish Embryos

Zebrafish (*Danio rerio*) were maintained at the University of Milan, Via Celoria 26—20133 Milan, Italy (Autorizzazione Protocollo n. 295/2012-A—20 December 2012). Zebrafish AB strains were maintained according to international (EU Directive 2010/63/EU) and national guidelines (Italian decree n. 26 of the 4 March 2014). Embryos were staged and used until five days post-fertilization, a time window in which zebrafish is not considered an animal model according to national guidelines (Italian decree n. 26 of the 4 March 2014). Embryos were staged according to Kimmel and colleagues [34] and raised in fish water (Instant Ocean, 0.1% Methylene Blue) at 28 °C in Petri dishes, according to established techniques. Embryonic ages are expressed in hours post-fertilization (hpf). Embryos were anesthetized with 0.016% tricaine (Ethyl 3-aminobenzoate methanesulfonate salt, Sigma-Aldrich) before proceeding with experimental protocols.

### 4.2. Injections

Injections were carried out on one- to two-cell stage embryos. Two different morpholinos specific for *nipblb*: the *nipblb*-ATG_MO (5′-GTCCCCATTCATGCTGAAGAAGGGA-3′) and the *nipblb*-5′UTR-MO (5′-TCGCTGCTACTGATCCACCTTTAC-3′, Gene Tools, LLC, Philomath, OR, USA) were injected together at the concentration of 0.5 pmol/embryo each as described in [21]. In all experiments, MO-injected embryos were compared to embryos at the same developmental stage injected with the same amount of a ctrl-MO that has no target in zebrafish (Gene Tools).

### 4.3. RNA Extraction

Total RNA was extracted from at least 30 embryos at the desired developmental stage (24 hpf) by TRIzol reagent (ThermoFisher Scientific, Waltham, MA, USA) and chloroform (Carlo Erba, Milan, Italy), followed by phenol-chloroform separation and isopropanol precipitation. The pellet was resuspended by adding 30 μL of RNAse-free water. Following RNA isolation, DNAse treatment was performed using Turbo DNAse (Life Technology, Carlsbad, CA, USA). After purification, RNA yield, quality, and size of isolated RNAs were assessed using Nanodrop spectrophotometer (ThemoFisher) and TapeStation instrument (Agilent Technologies, Santa Clara, CA, USA). Total RNA concentration from each biological sample ranged from 0.3 to 1.2 ug/μL, and the RNA Integrity Number (RIN) values were between 5.7 and 6.9.

### 4.4. RNA-Seq and Bioinformatics Analyses

Total RNA was isolated from ctrl-MO and *nipblb*-MO embryos at 24 hpf using the Illumina TruSeq Stranded mRNA Library Prep Kit, according to the manufacturer’s instructions. Before sequencing, libraries were analyzed with the TapeStation instrument and quantified by Nanodrop spectrophotometer with the use of the fluorescent dye PicoGreen^®^ (Thermo Fisher) to verify the length and concentration of the inserts. RNA libraries were then diluted at 2 nM concentration and normalized using standard library quantification and quality control procedures as recommended by the Illumina protocol. RNA sequencing was carried out in triplicates on an Illumina HiSeq4000 using a paired-end run (2 × 150 bases). Read quality was accessed using fastQC (v. 0.11.3; http://www.bioinformatics.babraham.ac.uk/projects/fastqc/). Raw reads were trimmed for adapters and for length at 100 bp with Trimmomatic [35] and subsequently aligned to the zebrafish reference genome (GRCz11.97) using STAR (v.020201, https://github.com/alexdobin/STAR) [36]. Raw gene counts were obtained using *htseq-count* function of HTSeq tool (v.0.6.0 with the *mode* option set as *reverse* for library type) [37] with the Ensembl annotation file GRCz11.97.gtf as a reference. Raw counts were normalized to counts per million mapped reads (cpm) using the *edgeR* package [38]. Only genes with a cpm greater than 1 in at least three samples were further retained for differential analysis. Global unsupervised clustering was performed using the function *hclust* of R *stats* package with Pearson correlation as distance metric and average agglomeration method. Gene expression heatmaps were generated using the function *heatmap.2* of R *gplots* package after row-wise standardization of the expression values. Before unsupervised clustering, to reduce the effect of noise from non-varying genes, we removed those probe sets with a coefficient of variation smaller than the 90th percentile of the coefficients of variation in the entire dataset. The filter retained 1922 genes that are more variable (highly variable genes) across samples in any of the two subsets (i.e., ctrl-MO and *nipblb*-MO at 24 hpf). Differential gene expression analysis was performed using the *exactTest* function of the *edgeR* package [38]. Genes were considered significantly differently expressed at False Discovery Rate (FDR) ≤ 0.01 and absolute fold-change ≥ 3. Functional annotation of the differentially expressed genes was performed using gProfiler (https://biit.cs.ut.ee/gprofiler) and the gene sets of the KEGG biological pathways. Functional over-representation analysis was performed using Gene Set Enrichment Analysis (GSEA; http://software.broadinstitute.org/gsea/index.jsp) and curated gene sets of the Molecular Signatures Database (MSigDB). In particular, we used the Hallmark gene sets, the KEGG subset of canonical pathways (MSigDB C2), and a collection of 80 gene sets related to myeloid differentiation and AML (Table S5). Prior to GSEA analysis, we converted zebrafish Entrez IDs into the corresponding human orthologues genes using the HUGO Gene Nomenclature Committee (HGNC) Orthology Predictions Search (HCOP; https://www.genenames.org/cgi-bin/hcop). Gene sets were considered significantly enriched at false discovery rate (FDR) ≤ 0.05 when using Signal2Noise as metric and 1.000 permutations of gene sets. The dot plots showing the most significantly enriched and depleted gene sets were generated using the *ggplot* function of the *ggplot2* R package.

Gene expression data of AML patients were measured on Affymetrix arrays and were downloaded from NCBI Gene Expression Omnibus (GEO, http://www.ncbi.nlm.nih.gov/geo/) GSE10358 along with annotations on the mutational status of NMP1. Since raw data (CEL files) were available for all samples, the integration, normalization, and summarization of gene expression signals were obtained by converting probe level signals to expression values using the robust multi-array average procedure RMA [39] of the Bioconductor *affy* package.

Average signature expression was calculated as the standardized average expression of all signature genes in all 184 samples with NMP1 annotation.

All analyses were performed using R 3.6.2 and publicly available packages explicitly cited in the manuscript. No custom functions were written for the analysis.

### 4.5. Data Availability

Raw reads of NGS data are available in NCBI Short-read Archive (SRA, https://www.ncbi.nlm.nih.gov/sra/) under accession number PRJNA675020.

## Supplementary Materials

Supplementary materials can be found at https://www.mdpi.com/1422-0067/21/24/9719/s1.

## Abbreviations

NIPBL	Nipped B-like
MO	Morpholino
RNA-seq	RNA sequencing
EMT	Epithelial–Mesenchymal Transition
ECM	Extracellular Matrix
AML	Acute Myeloid Leukemia
HSC	Hematopoietic Stem Cells
LSC	Leukemia Stem Cells
FDR	False Discovery Rate
GSEA	Gene Set Enrichment Analysis
NES	Normalized Enrichment Score
