# Supplementary material for: The Genome-Wide Impact of Nipblb Loss-of-Function on Zebrafish Gene Expression
Source: Int J Mol Sci. 2020 Dec 19;21(24):9719. doi: 10.3390/ijms21249719 (PMC7766774; doi:10.3390/ijms21249719)
Supplement: Supplementary file 1 [file ijms-21-09719-s001.zip › SpreaficoIJMS_Supplementary_Material_7Nov2020.pdf]

Table S1 Summary statistics of RNA-seq profiling and read alignment.

Sample	total_reads	mapped_reads	mapped_reads_pct
CTRL_24h_1	106320197	102654009	96.55
CTRL_24h_2	86519359	83030507	95.97
CTRL_24h_3	82622705	79702889	96.47
CTRL_3d_1	83993616	80332888	95.64
CTRL_3d_2	80608466	77092652	95.64
CTRL_3d_3	86163909	81937501	95.09
Nipblb_24h_1	78683922	75715162	96.23
Nipblb_24h_2	91981783	88171489	95.86
Nipblb_24h_3	58330315	56197319	96.34
Nipblb_3d_1	89482968	86230798	96.37
Nipblb_3d_2	74766515	72119105	96.46
Nipblb_3d_3	74548438	71882596	96.42

Table S2. Fold change (FC) and statistical significance (False Discovery Rate, FDR) of differential gene expression in the comparisons of *nipblb*-MO and ctrl-MO samples at 24 hpf and 3 dpf.

See Table S2.xlx

Table S3. Gene sets of the Hallmark and KEGG collections from the Molecular Signature Database (MSigDB; https://www.gsea-msigdb.org/gsea/msigdb/collections.jsp) enriched and depleted in *nipblb*-MO embryos as compared to ctrl-MO samples at 24 hpf (FDR≤0.05).

Gene set	Size	NES	FDR	GeneRatio	Effect
E2F TARGETS	170	3.58	0	0.69	Enriched in nipblb-MO
RIBOSOME	74	3.49	0	0.95	Enriched in nipblb-MO
MYC TARGETS V1	173	3.29	0	0.73	Enriched in nipblb-MO
DNA REPLICATION	29	3.18	0	0.97	Enriched in nipblb-MO
MYC TARGETS V2	54	3.07	0	0.85	Enriched in nipblb-MO
G2M CHECKPOINT	156	3.06	0	0.51	Enriched in nipblb-MO
HOMOLOGOUS RECOMBINATION	22	2.92	0	0.91	Enriched in nipblb-MO
SPLICEOSOME	102	2.89	0	0.74	Enriched in nipblb-MO
RNA POLYMERASE	22	2.82	0	0.91	Enriched in nipblb-MO
CELL CYCLE	94	2.81	0	0.59	Enriched in nipblb-MO
MISMATCH REPAIR	20	2.81	0	0.90	Enriched in nipblb-MO
PYRIMIDINE METABOLISM	72	2.73	0	0.63	Enriched in nipblb-MO
NUCLEOTIDE EXCISION REPAIR	36	2.66	0	0.83	Enriched in nipblb-MO
DNA REPAIR	120	2.62	0	0.56	Enriched in nipblb-MO
BASE EXCISION REPAIR	27	2.52	0	0.70	Enriched in nipblb-MO
RNA DEGRADATION	49	2.33	0	0.55	Enriched in nipblb-MO
PORPHYRIN AND CHLOROPHYLL METABOLISM	21	2.14	4.15E-04	0.43	Enriched in nipblb-MO
UNFOLDED PROTEIN RESPONSE	98	2.10	5.18E-04	0.41	Enriched in nipblb-MO
PROTEASOME	30	2.09	5.49E-04	0.73	Enriched in nipblb-MO
BASAL TRANSCRIPTION FACTORS	26	2.07	7.02E-04	0.69	Enriched in nipblb-MO
PROTEIN EXPORT	20	1.96	0.003	0.90	Enriched in nipblb-MO
AMINOACYL TRNA BIOSYNTHESIS	34	1.90	0.004	0.71	Enriched in nipblb-MO
CYTOSOLIC DNA SENSING PATHWAY	22	1.79	0.010	0.41	Enriched in nipblb-MO
P53 SIGNALING PATHWAY	50	1.75	0.012	0.42	Enriched in nipblb-MO
GLYCOSYLPHOSPHATIDYLINOSITOL GPI ANCHOR BIOSYNTHESIS	22	1.68	0.020	0.50	Enriched in nipblb-MO

NEUROACTIVE LIGAND RECEPTOR INTERACTION	123	-2.52	0	0.68	Enriched in CTRL-MO
CALCIUM SIGNALING PATHWAY	90	-2.42	0	0.64	Enriched in CTRL-MO
EPITHELIAL MESENCHYMAL TRANSITION	127	-2.29	0	0.54	Enriched in CTRL-MO
ECM RECEPTOR INTERACTION	51	-2.14	0	0.57	Enriched in CTRL-MO
LONG TERM POTENTIATION	34	-2.12	3.70E-04	0.65	Enriched in CTRL-MO
CARDIAC MUSCLE CONTRACTION	44	-2.10	6.07E-04	0.43	Enriched in CTRL-MO
TYPE II DIABETES MELLITUS	32	-2.07	7.95E-04	0.47	Enriched in CTRL-MO
MYOGENESIS	134	-2.06	8.16E-04	0.43	Enriched in CTRL-MO
GAP JUNCTION	50	-2.05	9.32E-04	0.72	Enriched in CTRL-MO
DILATED CARDIOMYOPATHY	57	-2.02	0.001	0.54	Enriched in CTRL-MO
OLFACTORY TRANSDUCTION	15	-2.01	0.001	1.00	Enriched in CTRL-MO
HYPERTROPHIC CARDIOMYOPATHY HCM	49	-2.00	0.001	0.47	Enriched in CTRL-MO
KRAS SIGNALING UP	117	-1.97	0.002	0.51	Enriched in CTRL-MO
FOCAL ADHESION	115	-1.94	0.002	0.41	Enriched in CTRL-MO
INOSITOL PHOSPHATE METABOLISM	36	-1.92	0.003	0.50	Enriched in CTRL-MO
PHOSPHATIDYLINOSITOL SIGNALING SYSTEM	47	-1.90	0.004	0.60	Enriched in CTRL-MO
GNRH SIGNALING PATHWAY	50	-1.88	0.004	0.64	Enriched in CTRL-MO
ARRHYTHMOGENIC RIGHT VENTRICULAR CARDIOMYOPATHY ARVC	47	-1.87	0.004	0.45	Enriched in CTRL-MO
CHEMOKINE SIGNALING PATHWAY	86	-1.84	0.006	0.58	Enriched in CTRL-MO
INFLAMMATORY RESPONSE	96	-1.82	0.007	0.36	Enriched in CTRL-MO
MAPK SIGNALING PATHWAY	148	-1.78	0.011	0.41	Enriched in CTRL-MO
INSULIN SIGNALING PATHWAY	82	-1.78	0.011	0.38	Enriched in CTRL-MO
APICAL JUNCTION	117	-1.77	0.011	0.37	Enriched in CTRL-MO
VASCULAR SMOOTH MUSCLE CONTRACTION	60	-1.77	0.010	0.50	Enriched in CTRL-MO
COMPLEMENT AND COAGULATION CASCADES	41	-1.76	0.012	0.66	Enriched in CTRL-MO
TRYPTOPHAN METABOLISM	25	-1.74	0.014	0.44	Enriched in CTRL-MO
ALDOSTERONE REGULATED SODIUM REABSORPTION	22	-1.72	0.016	0.68	Enriched in CTRL-MO
LONG TERM DEPRESSION	35	-1.69	0.024	0.54	Enriched in CTRL-MO
ESTROGEN RESPONSE EARLY	133	-1.68	0.023	0.46	Enriched in CTRL-MO
BILE ACID METABOLISM	82	-1.67	0.025	0.55	Enriched in CTRL-MO

CELL ADHESION MOLECULES CAMS	62	-1.64	0.033	0.37	Enriched in CTRL-MO
UV RESPONSE DN	97	-1.64	0.032	0.55	Enriched in CTRL-MO
AXON GUIDANCE	72	-1.63	0.034	0.65	Enriched in CTRL-MO
KRAS SIGNALING DN	97	-1.61	0.040	0.35	Enriched in CTRL-MO
AMYOTROPHIC LATERAL SCLEROSIS ALS	32	-1.60	0.043	0.44	Enriched in CTRL-MO

Table S4. Gene sets of the Hallmark and KEGG collections from the Molecular Signature Database (MSigDB; https://www.gsea-msigdb.org/gsea/msigdb/collections.jsp) enriched and depleted in *nipblb*-MO embryos as compared to ctrl-MO samples at 3 dpf (FDR≤0.05).

Gene set	Size	NES	FDR	GeneRatio	Effect
INTERFERON GAMMA RESPONSE	99	2.52	0	0.43	Enriched in nipblb-MO
RIBOSOME	74	2.44	0	0.76	Enriched in nipblb-MO
INTERFERON ALPHA RESPONSE	47	2.37	0	0.43	Enriched in nipblb-MO
PROTEASOME	30	2.16	7.249E-04	0.67	Enriched in nipblb-MO
IL6 JAK STAT3 SIGNALING	46	1.83	0.028	0.33	Enriched in nipblb-MO
DRUG METABOLISM CYTOCHROME P450	20	1.78	0.036	0.50	Enriched in nipblb-MO
AMINOACYL TRNA BIOSYNTHESIS	34	1.77	0.034	0.71	Enriched in nipblb-MO
STEROID HORMONE BIOSYNTHESIS	21	1.77	0.032	0.29	Enriched in nipblb-MO
METABOLISM OF XENOBIOTICS BY CYTOCHROME P450	22	1.73	0.042	0.45	Enriched in nipblb-MO
AXON GUIDANCE	72	-2.28	0	0.63	Enriched in CTRL-MO
NEUROACTIVE LIGAND RECEPTOR INTERACTION	123	-2.02	0.009	0.41	Enriched in CTRL-MO
CALCIUM SIGNALING PATHWAY	90	-1.97	0.011	0.37	Enriched in CTRL-MO
ECM RECEPTOR INTERACTION	51	-1.84	0.036	0.37	Enriched in CTRL-MO
NATURAL KILLER CELL MEDIATED CYTOTOXICITY	36	-1.82	0.037	0.36	Enriched in CTRL-MO
VEGF SIGNALING PATHWAY	31	-1.80	0.037	0.32	Enriched in CTRL-MO
FC GAMMA R MEDIATED PHAGOCYTOSIS	57	-1.76	0.044	0.40	Enriched in CTRL-MO

Table S5. Gene sets related to myeloid differentiation and AML derived from the Molecular Signature Database (MSigDB; https://www.gsea-msigdb.org/gsea/msigdb/collections.jsp).

See Table S5.xlx

Table S6. Gene sets related to myeloid differentiation and AML enriched and depleted in nipblb-MO embryos as compared to ctrl-MO samples at 24 hpf (FDR≤0.05).

Gene set	Size	NES	FDR	GeneRatio	Effect
EPPERT_PROGENITOR	114	1.83	0.039	0.49	Enriched in nipblb-MO
GAL_LEUKEMIC_STEM_CELL_DN	127	1.71	0.046	0.37	Enriched in nipblb-MO
ALCALAY_AML_BY_NPM1_LOCALIZATION_DN	131	1.67	0.041	0.37	Enriched in nipblb-MO
EPPERT_HSC_R	80	-1.98	0.005	0.44	Enriched in CTRL-MO
VERHAAK_AML_WITH_NPM1_MUTATED_DN	147	-1.98	0.003	0.52	Enriched in CTRL-MO
EPPERT_CE_HSC_LSC	22	-1.88	0.007	0.55	Enriched in CTRL-MO
VALK_AML_CLUSTER_7	15	-1.81	0.016	0.80	Enriched in CTRL-MO
VALK_AML_CLUSTER_16	19	-1.80	0.013	0.53	Enriched in CTRL-MO
VALK_AML_CLUSTER_11	23	-1.75	0.019	0.30	Enriched in CTRL-MO
VALK_AML_CLUSTER_10	21	-1.71	0.023	0.76	Enriched in CTRL-MO
